# Bioactive Compounds, Antioxidant, and Antimicrobial Activity of Seeds and Mucilage of Non-Traditional Cocoas

**DOI:** 10.3390/antiox14030299

**Published:** 2025-02-28

**Authors:** Elena Coyago-Cruz, Iván Salazar, Aida Guachamin, Melany Alomoto, Marco Cerna, Gabriela Mendez, Jorge Heredia-Moya, Edwin Vera

**Affiliations:** 1 Carrera de Ingeniería en Biotecnología de los Recursos Naturales, Universidad Politécnica Salesiana, Sede Quito, Campus El Girón, Av. 12 de Octubre N2422 y Wilson, Quito 170109, Ecuador; 2 Maestría en Productos Farmacéuticos Naturales, Universidad Politécnica Salesiana, Sede Quito, Campus El Girón, Av. 12 de Octubre N2422 y Wilson, Quito 170109, Ecuador; 3 Centro de Investigación Biomédica (CENBIO), Facultad de Ciencias de la Salud Eugenio Espejo, Universidad UTE, Quito 170527, Ecuador; 4 Departamento de Ciencia de los Alimentos y Biotecnología, Facultad de Ingeniería Química, Escuela Politécnica Nacional, Quito 170524, Ecuador

**Keywords:** carotenoids, phenols, organic acid, microextraction, in vitro, Amazon rainforest

## Abstract

The biodiversity of the Amazon rainforest includes little-known cocoa species, which are essential resources for local communities. This study evaluated the bioactive compounds and antioxidant and antimicrobial activity of seeds and mucilage of four non-traditional cocoa species (*Theobroma subincanum*, *T. speciosum*, *T. bicolor* and *Herrania nitida*). Physico-chemical properties, minerals, vitamin C, organic acids, phenolics, and carotenoids were analysed by spectrophotometric and chromatographic techniques. The antioxidant activity was measured by ABTS and DPPH, along with the antimicrobial activity against *Escherichia coli*, *Staphylococcus aureus*, *Pseudomonas aeruginosa*, and *Streptococcus mutans*, as well as *Candida albicans* and *Candida tropicalis*. *T. subincanum* seeds scored high in titratable acidity, magnesium, sodium, syringic acid, chlorogenic acid, caffeic acid, rutin, and quercetin. In contrast, the mucilage scored high in calcium, *m*-coumaric acid, ferulic acid, kaempferol, quercetin glycoside, and antimicrobial activity against *Streptococcus mutans. T. speciosum* mucilage excelled in malic acid, tartaric acid, naringenin, and antioxidant capacity. *T. bicolor* seeds excelled in lutein and antimicrobial activity against *Staphylococcus aureus* and *Candida albicans*, and mucilage in iron, potassium, vitamin C, citric acid, gallic acid and 4-hydroxybenzoic acid, zeaxanthin, β-carotene, and antioxidant capacity by ABTS. The mucilage of *H. nitida* has a high soluble solids content. These results highlight the potential of these species as sustainable sources of functional compounds and nutraceuticals.

## 1. Introduction

The Amazon rainforest, recognised as one of the most biodiverse regions on the planet, is home to a wide range of plant species with remarkable medicinal and nutritional properties. Among these is the genus *Theobroma*, which belongs to the Malvaceae family, which includes 246 genera with more than 4225 species, some of which are important for their economic and cultural value [1]. This genus contains species native to the Amazon region that are valuable for sustaining local economies and play a key role in the conservation of tropical ecosystems by indigenous communities in Latin America and other tropical regions of Asia and Africa [2].

The term Theobroma, which means ‘food of the gods’ in Greek, reflects these species’ historical and cultural importance [3]. Pre-Columbian cultures such as the Maya and Aztecs have valued cacao (*T. cacao*) for over 2500 years. Initially found in the river valleys of South America, cacao trees were introduced to Mexico by the Maya Indians in the 7th century AD and were also cultivated by the Aztecs and Toltecs. Ancient texts describe various cocoa blends used for ceremonial, medicinal, and culinary purposes [4].

Over the centuries, these practices have evolved into a multi-billion dollar global market that includes chocolate and a wide variety of cocoa-derived products [5]. Globally, *T. cacao*, commonly known as cocoa, is widely cultivated, with varieties such as ‘Nacional’, ‘Criollo’, ‘Forastero’, and ‘Trinitario’, each with specific characteristics that respond to market needs and growing conditions [3,6]. For example, the ‘Nacional’ variety, grown mainly in Ecuador, is recognised for its quality and flavour, while the CCN-51 clone was developed to improve yield and disease resistance [7]. Global demand for chocolate and cocoa products drives a strong international market for cocoa beans. It is an important source of income for millions of smallholder farmers in tropical regions, mainly in West Africa, Latin America, and Southeast Asia [4,7,8]

Although *Theobroma cacao* is the species best known for its use in chocolate production [9], other species such as *Theobroma bicolor* (macambo, white cacao, patashte, balmte, cacao malacayo, wild cacao, white cacao, maraco), *Theobroma grandiflorum* (copoazú), Theobroma subincanum (macambillo) [10], *Theobroma speciosum* (cacauhy, cacauo silveste) [11,12], *Theobroma subincana* (cacao de monte, sacha cacao, cupuí, macamillo) [13,14], and related species such as *Herrania nitida* (wild cacao, cacao de monte, monkey cacao) [3,15] are used locally in the production of products such as juices, ice cream, and liqueurs, highlighting their versatility and economic importance for the sectors that cultivate them [2]. These species, which are currently in the process of domestication and industrialisation, have limited scientific studies [2,16,17].

The seeds and other tissues of *Theobroma* spp. and *Herrania* contain many bioactive compounds, including alkaloids, organic acids, flavonoids, and fatty acids such as palmitic, oleic, and linoleic acids. Phenolic compounds such as catechins, proanthocyanidins, and anthocyanins stand out for their antioxidant capacity. These compounds are key in neutralising free radicals and reducing oxidative stress, contributing to cardioprotective, neuroprotective, and hepatoprotective effects [18,19]. In addition, studies have shown that consuming products rich in cocoa polyphenols can attenuate obesity and reduce insulin resistance and markers of metabolic diseases such as fatty liver [5]. For example, studies show that *T. grandiflorum* and *T. bicolor* pulp are consumed fresh, while their seeds, rich in phenolic compounds, are roasted. In addition, extracts of these species have shown cytotoxic and antimicrobial activities, including the inhibition of *Escherichia coli. T. grandiflorum* pulp meals were shown to have dietary antioxidants and prebiotic, immunomodulatory, and anti-inflammatory properties. Similarly, *T. speciosum*, although less studied, is considered a valuable source of pest-resistant genes within the genus *Theobroma* [2,10].

In this context, the present study aimed to investigate the bioactive compounds and the antioxidant and antimicrobial activities of the seeds and mucilage of non-traditional cocoas. This approach highlights the potential health benefits associated with consuming these cocoa species, emphasising their potential as a source of nutraceuticals and functional ingredients. Although some bioactive compounds were already identified, significant knowledge gaps exist, especially in the detailed characterisation of secondary metabolites. It is, therefore, essential to carry out more in-depth and systematic research to improve the understanding of the bioactive composition and explore its viability and applications in the global market as innovative and sustainable ingredients.

## 2. Materials and Methods

### 2.1. Reagents and Standards

The chemical substances employed throughout this investigation were acetone (CAS67-64-1) and fluconazole (CAS86386-73-4), classified as reagent-grade quality. Additionally, the high-performance liquid chromatography (HPLC) grade chemicals that were utilised in this study encompassed acetonitrile (CAS 75-05-8), ethanol (CAS 64-17-5), and methanol (CAS 67-56-1), all of which were procured from Fisher Chemical (Fischer Scientific Inc., Madrid, Spain). Additionally, a variety of other analytical grade chemicals were employed, specifically ABTS (2,2′-azino-bis-(3-ethylbenzothiazoline-6-sulfonic acid) (CAS30931-67-0), DPPH (2,2-Diphenyl-1-picrylhydrazyl (CAS1898-66-4), formic acid (CAS 64-18-6), Folin–Ciocalteu reagent (CAS 7732-18-5), metaphosphoric acid (CAS 37267-86-0), nitric acid (CAS 7697-37-2), potassium chloride (CAS 7447-40.7), potassium persulphate (CAS 7727-21-1), sodium acetate trihydrate (CAS 6131-90.4), sodium carbonate (CAS 497-19-8), and sodium hydroxide (CAS 1310-73-2); all of these analytical grade chemicals were sourced from Sigma, a division of Merck, located in Darmstadt, Germany. Furthermore, hydrochloric acid (CAS 7647-01-0) was also acquired in its analytical grade form from Labscan (RCI Labscan, Dublin, Republic of Ireland). Various microbiological growth media, such as Brain Heart Infusion (BHI), Mueller Hinton agar (MHA), and Sabouraud dextrose agar (SDA), were obtained from BD DifcoTM (Fisher Scientific Inc., Madrid, Spain). Additionally, Yeast Peptone Dextrose Broth (YPDB) was sourced from SRL (Sisco Research Laboratories Pvt. Ltd., Mumbai, India), while streptomycin sulphate (CAS 3810-74-0) was obtained from Phytotech (PhytoTechnology Laboratories ^®^, Lenexa, KS, USA). The water utilised throughout the study was purified using a NANOpureDiamondTM system (Barnsted Inc., Dubuque, IA, USA).

The standards that were utilised within the scope of this investigation included β-carotene with a purity of 93.0% (CAS 7235-40-7), caffeic acid with a purity of 98.0% (CAS 331-39-5), chlorogenic acid with a purity of 95.0% (CAS 327-97-9), chrysin with a purity of 97.0% (CAS 480-40-0), cyanidin-e-glucoside chloride with a purity of 97.0% (CAS 7084-24-4), ferulic acid with a remarkable purity of 100.0% (CAS 1135-24-6), and gallic acid, which also exhibited a purity of 100.0% (CAS 149-91-7). Other standards included 2,5-dihydroxybenzoic acid with a purity of 98.0% (CAS 490-79-9), 3-hydroxybenzoic acid with a purity of 99.0% (CAS 99-06-3), kaempferol with a purity of 97.0% (CAS 520-18-3), luteolin with a purity of 98% (CAS 491-70-3), *m*-coumaric acid with a purity of 99.0% (CAS 588-30-7), naringin with a purity of 95.0% (CAS 10236-47-2), o-coumaric acid with a purity of 97.0% (CAS 614-60-8), *p*-coumaric acid with a purity of 98.0% (CAS 501-98-4), *p*-hydroxybenzoic acid with a purity of 99.0% (CAS 99-06-3), quercetin with a purity of 95.0% (CAS 849061-97-8), rutin with a purity of 94.0% (CAS 153-18-4), shikimic acid with a purity of 99.0% (CAS 138-59-0), syringic acid with a purity of 95.0% (CAS 530-57-4), vanillic acid with a purity of 97.0% (CAS 121-34-6), and Trolox with a purity of 98% (CAS 53188-07-1); all of these standards were acquired from Sigma, a division of Merck, located in Darmstadt, Germany. In turn, calcium (CAS7440-70-2), iron (CAS 7439-89-6), magnesium (CAS7439-95-4), potassium (CAS7440-09-7), and sodium (CAS7440-23-5) with a concentration of 1000 µg/mL were purchased from Accustandard (AccuStandard, Inc, New Haven, CT, USA). The microbial strains employed in this study included *Candida albicans* ATCC 1031, *Candida tropicalis* ATCC 13803, *Escherichia coli* ATCC 8739, *Pseudomonas aeruginosa* ATCC 9027, *Staphylococcus aureus* ATCC 6538P, and *Streptococcus mutans* ATCC 25175, all of which were procured from the American Type Culture Collection (ATCC), based in Manassas, VA, USA.

### 2.2. Physico-Chemical Analyses

**Figure 1:**
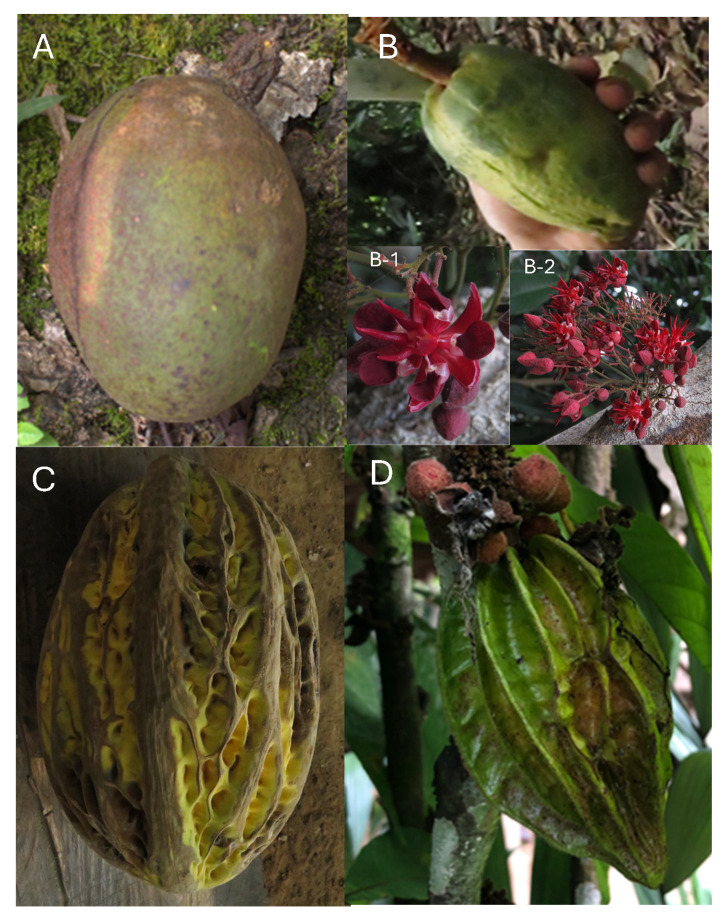
Photograph of different types of cocoa. Note: (**A**) *Theobroma subincanum* Mart. (Identification code: 4747, Herbario QUPS-Ecuador); (**B**) *Theobroma speciosum* Wild. Ex Spreng (Identification code: 4751, Herbario QUPS-Ecuador); (**B-1**,**B-2**) *T. speciosum* flowers; (**C**) *Theobroma bicolor* Bonpl. (Identification code: 4752, Herbario QUPS-Ecuador); (**D**) *Herrania nitida* (Poepp.) R.E.Schult. (Identification code: 4784, Herbario QUPS-Ecuador).

The study included ripe fruit (visual appearance) without defects from four different cocoa species such as *Theobroma subincanum*, *Theobroma speciosum*, *Theobroma bicolor*, and *Herrania nitida*. These species were purchased in the Ecuadorian Amazon province of Pastaza (1°44′21″ S, 77°29′1″ W). In addition, plant samples were collected for identification at the herbarium of the Universidad Politécnica Salesiana (Figure 1).

Samples were randomly selected, and five fruits were considered for physico-chemical analysis. This analysis included the determination of the weight and size (equatorial and longitudinal diameter) of the fruits and their seeds. In turn, the collected pulp and seeds were separately homogenised, and a portion was taken for fresh measurements. These measurements included pH using a SevenMultiS47 (Mettler Toledo, Columbus, OH, USA); soluble solids using a Hitech RHB-32 refractometer (G-Won Hitech Co., Lt., Seoul, Republic of Korea) following the ISO 2173 methodology [20]; total titratable acidity by acid-base titration; moisture content using a Memmert Be 20 oven (Memmert GmbH + Co.KG, Barcelona, Spain) at 100 °C; and ash determination by calcination in a muffle (Thermo Fisher Scientific, Waltham, MA, USA) at 550 °C. The other part of the samples was frozen at −80 °C and lyophilised in a Christ Alpha 1-4 LDplus (GmbH, Osterode am Harz, Germany). The dried samples were crushed and stored in amber vials under a nitrogen atmosphere until analysis [21].

#### Mineral Profile

For mineral extraction, 40 mg of lyophilized powder was weighed into a Speedwave Xpert microwave Teflon digester (Berghof products + Instruments GmbH, Eningen unter Achalm, Germany) and mixed with 5 mL of 65% nitric acid. The mixture was allowed to stand for 10 min before the digester was closed. Digestion was performed with a linear gradient of 140 °C, 30 bar, 70% power for 5 min; 200 °C, 35 bar, 80% power for 15 min; and 50 °C, 25 bar, 0% power for 10 min. The digesters were allowed to cool at room temperature for 20 min [22]. The digested sample was made up to 25 mL with MilliQ water and stored in amber glass vials until analysis.

Mineral quantification was performed using a Varian SpectrAA-55 atomic absorption spectrophotometer. Calcium was determined at 422.7 nm with a slit width of 0.5 and an acetylene-nitrous oxide mixture. In contrast, the following minerals were determined with an air–acetylene mixture, i.e., iron at a wavelength of 372.0 nm and a slit width of 0.20 nm, sodium at 589.6 nm and 0.5 nm, potassium at 404.4 nm and 0.5 nm, and magnesium at 202.6 nm and 1.0 nm. The concentration of the standard solution was 1000 ppm, and solutions were prepared between 0 and 5 ppm for Ca, 0 and 20 ppm for Fe, 0 and 200 ppm for K, 0 and 10 ppm for Mg, and 0 and 8 for Na. The concentration was expressed in milligrams of mineral per 100 g dry weight (mg/100 g DW).

### 2.3. Analysis of Bioactive Compounds

#### 2.3.1. Vitamin C

The quantification of vitamin C (ascorbic acid) was performed according to the protocol described by Coyago et al. [18]. Briefly, 40 mg of lyophilised powder was mixed with 1.2 mL of a 3% aqueous metaphosphoric acid solution. The mixture was homogenised using a VM-300 vortex mixer (Interbiolab Inc., Orlando, FL, USA) and stirred in an FS60 ultrasonic bath (Fisher Scientific Inc., Waltham, MA, USA). The volume was then made up to 2 mL with Milli-Q water.

The resulting extract was filtered and analysed on a 1200 RRLC high-performance liquid chromatograph (Agilent Technologies, Mississauga, ON, Canada) equipped with a DAD-UV-VIS detector and a ZORBAX Eclipse XDB 80 AC C18 column (1.8 µm, 4.6 mm × 50 mm) (Agilent Scientific Instruments, Santa Clara, CA, USA). An ascorbic acid standard was used, and the concentration was expressed as milligrams of ascorbic acid per 100 g dry weight (mg/100 g DW).

#### 2.3.2. Organic Acid Profile

The individual organic acids were determined as described by Coyago et al. [18]. A 40 mg lyophilised powder mixture was prepared with 1.5 mL of 0.02 N sulphuric acid containing 0.05% metaphosphoric acid and 0.02% homocysteine. The mixture was homogenised in a VM-300 vortex mixer (Interbiolab Inc, Orlando, FL, USA) and vortexed in an FS60 ultrasonic bath (Fisher Scientific Inc., Waltham, MA, USA). The liquid extract was filtered and quantified on a 1200 RRLC liquid chromatograph (Agilent Technologies, Mississauga, ON, Canada) equipped with a DAD-UV-VIS detector and a YMC-Triart C18 column (3 µm, 4.6 mm × 150 mm) (YMC Europe GmbH, Dinslaken, Germany). Citric, malic, and tartaric acid standards were used. Concentrations were expressed as milligrams of organic acid per 100 g dry weight (mg/100 g DW).

#### 2.3.3. Carotenoid Profile

A 20 mg lyophilised powder mixture was prepared with 250 µL methanol, 500 µL chloroform, and 250 µL MilliQ water. The mixture was homogenised in a VM-300 vortex mixer (Interbiolab Inc, Orlando, FL, USA) and vortexed in an FS60 ultrasonic bath (Fisher Scientific Inc, Waltham, MA, USA). This process was repeated until all colour was extracted from the solid. The coloured phase was collected and evaporated to dryness in a vacuum rotary evaporator below 30 °C [18].

Carotenoid quantification was performed as described by Coyago et al. [23]. The dried extract was redissolved with 20 µL ethyl acetate and quantified on a 1200 RRLC liquid chromatograph (Agilent Technologies, Mississauga, ON, Canada) equipped with a DAD-UV-VIS detector and a YMC C30 column (3 µm, 4.6 × 150 mm) (YMC Europe GmbH, Dinslaken, Germany). Standards for astaxanthin, α-carotene, β-carotene, β-cryptoxanthin, lycopene, lutein, trans-β-apo-8-carotenal, violaxanthin, and zeaxanthin were used. Total carotenoids were calculated as the sum of the individual carotenoids expressed in milligrams of carotenoid per 100 g dry weight (mg/100 g DW).

#### 2.3.4. Phenol Profile

The individual phenolic compounds were determined as described by Coyago et al. [18]. A 20 mg lyophilised powder mixture was prepared with 500 µL of an aqueous solution of 80% methanol acidified with 0.1% HCl. The mixture was homogenised in a VM-300 vortex mixer (Interbiolab Inc., Orlando, FL, USA) and stirred in an FS60 ultrasonic bath (Fisher Scientific Inc., Waltham, MA, USA). The methanolic phase was filtered and quantified on a 1200 RRLC liquid chromatograph (Agilent Technologies, Mississauga, ON, Canada) equipped with a DAD-UV-VIS detector and a ZORBAX Eclipse Plus C18 column (5 µm, 4.6 mm × 150 mm) (Agilent Scientific Instruments, Santa Clara, CA, USA). Standards of shikimic acid, benzoic acids (*p*-hydroxybenzoic acid, 3-hydroxybenzoic acid, 2-methoxy benzoic acid, 3-methoxy benzoic acid, 3-hydroxybenzoic acid, 2,5-dihydroxybenzoic acid, gallic acid, protocatechuic acid, vanillic acid, syringic acid, ellagic acid, tannic acid, *p*-coumaric acid, *m*-coumaric acid, *o*-coumaric acid), hydroxycinnamic acids (chlorogenic, caffeic, ferulic), flavonoids (rutin, quercetin, myricetin, kaempferol, quercetin glycoside), flavones (chrysin, luteolin, rutin), flavanols (catechin, epicatechin), and flavonoids (naringenin, naringin) were utilised. Total phenolics were calculated as the sum of individual phenolics expressed in milligrams of phenolic compound per 100 g dry weight (mg/100 g DW).

### 2.4. Antioxidant Activity Analyses

Antioxidant activity was determined by the ABTS and DPPH methods described by Coyago and coworkers [24]. For ABTS quantification, a 20 mg lyophilised powder mixture was prepared with 400 µL methanol and 400 µL distilled water. For quantification by DPPH, a mixture of 20 mg lyophilised powder was prepared with 2 mL methanol. The mixture was homogenised in a Vortex Mixer VM-300 (Interbiolab Inc., Orlando, FL, USA) and stirred in an FS60 ultrasonic bath (Fisher Scientific Inc., Waltham, MA, USA). The liquid phase was reacted with ABTS•+ or DPPH− radical (10), and the absorbance was determined on a Multiskan GO microplate reader spectrophotometer (Agilent Scientific Instruments, Santa Clara, CA, USA). A Trolox standard was used, and the concentration was expressed as mmol Trolox equivalents per 100 g dry weight (mmol TE/100 g DW).

### 2.5. Antimicrobial Activity Analyses

Antimicrobial activity was determined as described by Coyago et al. [23]. The dry extract was prepared by mixing 2 g of lyophilised powder with 10 mL of 50% ethanol and then evaporating to dryness by lyophilisation. The antibacterial activity was evaluated against *Staphylococcus aureus* ATCC 6538P, *Escherichia coli* ATCC 8739, *Pseudomonas aeruginosa* ATCC 9027, and *Streptococcus mutans* ATCC 25175. The antifungal activity was tested against *Candida albicans* ATCC 1031 and *Candida tropicalis* CC 13803. A suitable diffusion method was used to evaluate antimicrobial activity. The inhibition zone was measured in mm. Streptomycin and fluconazole were used as positive controls for bacteria and fungi, respectively, and water was used as the negative control.

### 2.6. Statistical Analysis

STATGRAPHICS CenturionXVII, Rstudio 4.3.3 and SigmaPlot 14.0 were used for statistical analysis. The results were expressed as mean + standard deviation. One-way ANOVA was used. Tukey’s test performed a separation of means with significant differences at 0.05, and Pearson correlations were used at a 95% confidence level to estimate the differences between the pulp and the seeds within the same species, as well as between the pulp and the seeds of different species. In addition, principal component analysis (PCA) was used to select the variables that most influenced the differences between cocoa species. In this analysis, all the variables evaluated were compared, including carotenoids, phenolics, and total organic acid; bioactive compounds and antioxidant activity using ABTS and DPPH; antimicrobial activity; and the complete set of parameters analysed. As the variables had different units of measurement, a standardisation process was carried out in which the values were centred on their mean and scaled to one unit of variance. This ensured that all variables had a mean of 0 and a variance of 1, preventing those with larger magnitudes from dominating the analysis and ensuring an equitable contribution of each variable to the model.

## 3. Results and Discussion

### 3.1. Physico-Chemical Characteristics

The study included ripe fruits from four different species of *Theobroma cacao*, a species widely known worldwide for its properties and uses in chocolate production, as well as for its benefits. However, other species, such as *T. bicolor*, *T. speciosum*, *T. subincanum*, and *Herrania nitida*, are native to South America, and very little is known about their beneficial properties [25,26,27]. The results of the physical and chemical characteristics of the fruits studied are presented below.

#### 3.1.1. Weight and Size

Table 1 shows the average weight and size of the cacao species studied. A wide variability in fruit weight was observed, ranging from very light species, such as *Herrania nitida*, to considerably heavier species, such as *Theobroma bicolor*. Fruit size also varied considerably. The longitudinal diameter ranged from 21.8 mm (*T. speciosum*) to 470.0 mm (*T. bicolor*), while the equatorial diameter ranged from 12.4 mm (*T. speciosum*) to 303.3 mm (*T. bicolor*). Seed weights ranged from 1.3 g (*H. nitida*) to 5.4 g (*T. bicolor*). Seed sizes also varied considerably, with longitudinal diameters ranging from 0.3 mm (*T. speciosum*) to 1.9 mm (*T. bicolor*) and equatorial diameters ranging from 0.4 mm to 1.6 mm in the same species.

In this study, the weights of *T. subincanum* and *T. speciosum* were comparable to those previously reported by other authors, with 164.87 g and 84.08 g, respectively, in species native to the Brazilian Amazon [28]. The weights of *T. bicolor* cobs in this study were higher than those recorded for species cultivated in Colombia (300.0 to 400.0 g) [29]. However, they remained within the range reported for species cultivated on the Ecuadorian coast (714 to 1443 g) [30]. As for the seeds of *T. bicolor*, their weight was lower than that documented on the Ecuadorian coast (9 g) but similar to that of *T. speciosum* and higher than that of *T. subincanum*. The latter had weights of 1.6 g and 1.28 g, respectively, according to studies from the Ecuadorian Amazon [28].

Regarding size, *T. bicolor* fruits had larger longitudinal diameters than previously reported (21.0 to 25.3 cm) and larger equatorial diameters ranging from 10.7 to 11.5 cm [30]. These differences could be attributed to climatic conditions specific to the growing areas, such as solar radiation, rainfall, temperature, and altitude, which influence fruit development and ripening [31].

#### 3.1.2. pH

Table 2 shows the results of the chemical characteristics analysed, including pH, soluble solids, titratable acidity, moisture content, ash, and minerals (calcium, iron, potassium, magnesium, and sodium) in the seeds and mucilage of the cocoa species studied. The pH of the mucilage ranged from 4.6 (*H. nitida*) to 6.4 (*T. speciosum*), while the pH of the seeds ranged from 3.7 (*T. subincanum* and *T. speciosum*) to 6.7 (*T. bicolor*). The soluble solids in the mucilage ranged from 7.7 °Brix (*T. bicolor*) to 12.0 °Brix (*H. nitida*), while in the seeds, they ranged from 6.0 °Brix (*T. bicolor*) to 10.0 °Brix (*H. nitida*).

pH showed significant variation between cultivars and the components analysed (mucilage and seeds). These results are in agreement with previously reported values, such as a pH of 6.0 in fresh seeds of *T. bicolor* [32], 6.03 in dry seeds [30], and a range of 6.0 to 6.5 in mucilage from fruits 83 to 113 days old [29]. The cacao seeds’ pH and mucilage vary considerably due to factors such as fermentation and pod storage. In *T. cacao*, the pulp generally has a lower pH than the seeds, which is influenced by converting sugars to organic acids during fermentation. Although pH is mainly a function of enzymatic and fermentation processes, it can also be influenced by regional factors and specific processing methods. In addition, the acidity of the mucilage is vital to facilitate fermentation, an essential step in the development of cocoa’s characteristic flavour compounds. However, excessive acidity can interfere with this process [33,34].

#### 3.1.3. Soluble Solids and Titratable Acidity

Soluble solids ranged from 6.0 °Brix in *T. bicolor* seeds to 12.0 °Brix in *H. nitida* mucilage. These values are comparable to those reported in previous studies, which document a range of 4.4 to 11.0 in the mucilage of fruits with 83 to 113 days of development [29]. The differences observed in cocoa bean soluble solids and mucilage are significantly influenced by variety. Mucilage, often considered a by-product, contains a remarkable concentration of soluble solids, which play an essential role in the fermentation process and the development of flavour profiles. In *T. cacao*, this concentration reaches approximately 14.8 °Brix [35], exceeding the values observed in the species studied. Thus, the high soluble solids content of the mucilage also represents a valuable resource for the development of functional products, such as probiotic beverages. Furthermore, differences in sugar content between species can influence fermentation times and flavour development, which are essential factors for the final quality of chocolate.

On the other hand, the titratable acidity reached its highest values in the seeds of *T. subincanum* (1.7%) and *H. nitida* (0.8%), which are results in agreement with studies reporting an acidity of 0.45% in *T. bicolor* [30]. In the mucilage, the titratable acidity ranged from 0.1% (*T. speciosum*) to 0.6% (*T. subincanum* and *T. bicolor*), while in the seeds, it ranged from 0.4% (*T. bicolor*) to 1.7 (*T. subincanum*).

#### 3.1.4. Moisture and Ash

Moisture showed significant differences between the species studied. In the mucilage, it varied from 39.5% in *T. speciosum* to 96.0% in *T. bicolor*, while in the seeds, it varied from 37.6% in *H. nitida* to 92.7% in *T. bicolor*. *T. bicolor* showed the highest values for both mucilage (96.0%) and seeds (92.7%), which could affect its texture and stability. These results exceed previously reported values, such as 3.6% and 3.5% for the dry seeds of *T. bicolor* [30,32], and 87.9% for mucilage [29].

As for the ash content, the values for the mucilage ranged from 1.2% (*T. speciosum* and *H. nitida*) to 4.2% (*T. bicolor*), while the values for the seeds ranged from 1.7% (*T. bicolor*) to 2.6% (*T. subincanum*, *T. speciosum* and *H. nitida*). This reflects higher mineral content in these species. The results are comparable to those reported in previous studies, which documented 3.5% ash in *T. bicolor* seeds [30,32] and up to 8.2% in the mucilage [29].

#### 3.1.5. Minerals

Regarding minerals, potassium had the highest values in mucilage and seeds. Calcium in mucilage ranged from 276.4 mg/100 g DW (*T. bicolor*) to 1304.1 mg/100 g DW (*T. subincanum*), while in the seeds, it ranged from 126.4 (*T. speciosum*) to 752.1 mg/100 g DW (*T. subincanum*). Iron in the mucilage ranged from 46.8 mg/100 g DW (*T. speciosum*) to 77.0 mg/100 g DW (*T. bicolor*), while in the seeds, it ranged from 12.4 mg/100 g DW (*H. nitida*) to 44.6 mg/100 g DW (*T. subincanum*). Potassium in the mucilage ranged from 41342.6 mg/100 g DW (*T. subincanum*) to 595.2 mg/100 g DW (*T. bicolor*), while in the seeds, it ranged from 774.4 mg/100 g DW (*H. nitida*) to 1896.6 mg/100 g DW (*T. speciosum*). Magnesium in the mucilage ranged from 160.7 mg/100 g DW (*T. speciosum*) to 314.6 mg/100 g DW (*T. subincanum*), while in the seeds, it ranged from 238.7 mg/100 g DW (*T. bicolor*) to 382.7 mg/100 g DW (*T. subincanum*). Finally, the sodium content of the seeds ranged from 2.7 mg/100 g DW (*T. speciosum*) to 59.3 mg/100 g DW (*T. subincanum*).

In this sense, calcium is a mineral that strengthens the cell wall structure, extending the shelf life of the fruit by reducing the activity of cell wall degrading enzymes and delaying softening. In addition, calcium reduces ethylene production, delaying the peak of the climacteric and associated ripening processes, helping to preserve fruit quality, flavour, and nutritional value [36]. Nutritionally, adults’ recommended daily calcium intake is between 700 and 1200 mg. Depending on age, gender, and health conditions, it is essential to maintain bone health and prevent diseases such as osteoporosis [37].

Iron content was remarkably high in *T. bicolor* mucilage (77.0 mg/100 g DW) and *T. subincanum* seeds (44.6 mg/100 g DW), positioning them as potential dietary sources of this essential mineral. Iron is essential for key biological functions, and its presence in fruits can supplement their intake as part of a balanced diet. Although concentrations vary between fruits, some, such as peaches, are particularly rich in iron [38].

Regarding potassium, *T. bicolor* had the highest concentrations in the mucilage (4595.2 mg/100 g DW), while *T. speciosum* had higher concentrations in the seeds (1896.6 mg/100 g DW). Potassium, essential for muscle function and electrolyte balance, is mainly found in fruits, vegetables, roots, and tubers. Globally, dietary potassium intake has increased from 2984 mg per capita per day in 1961 to 3796 mg in 2017, reflecting increased availability [39]. These differences in potassium content between fruits allow dietary recommendations to be adjusted, especially for individuals who need to control sodium intake or suffer from hypertension [40].

Magnesium was high in *T. subincanum* seeds (382.7 mg/100 g DW) and mucilage (314.6 mg/100 g DW). This mineral is essential for many metabolic functions and contributes to the daily dietary intake. Although magnesium is generally lower in fruits compared to sources such as legumes and vegetables, its concentration may vary due to environmental and agronomic factors, including the effects of climate change [41].

Finally, sodium levels were highest in the mucilage (54.8 mg/100 g DW) and seeds (59.3 mg/100 g DW) of T. subincanum, exceeding the other cultivars. Although fruits are not a significant source of sodium, their sodium content is generally low compared to processed foods. In terms of diet, the recommended daily intake of sodium for adults is approximately 2300 mg, depending on individual needs and specific health guidelines [42,43]

### 3.2. Analysis of Bioactive Compounds

Cocoa (*T. cacao*) is widely known for its bioactive compounds and numerous health benefits. However, little information on related species such as *T. bicolor*, *T. speciosum*, *T. subincanum*, and *Herrania nitida* is available. Thus, Table 3 shows the results regarding bioactive compounds, including vitamin C, organic acids, phenolic compounds, carotenoids, and antioxidant activity.

#### 3.2.1. Vitamin C

As a potent antioxidant, vitamin C scavenges reactive oxygen and nitrogen species, protecting cells from oxidative damage, reducing inflammation and helping to prevent chronic diseases [44]. Thus, the concentration of vitamin C in the mucilage ranged from 3.8 mg/100 g DW (*T. subincanum*) to 70.5 mg/100 g DW (*T. bicolor*). In contrast, the seeds ranged from no detectable values (*T. subincanum*) to 55.1 mg/100 g DW (*T. speciosum*). These values are considerably higher than those reported in other studies, e.g., 0.001 mg/100 g in *T. bicolor* mucilage [29].

#### 3.2.2. Organic Acids

In the case of organic acids, the concentration of citric acid in the mucilage ranged from 112.4 mg/100 g dry weight (*T. subincanum*) to 15557.9 g/100 g dry weight (*T. bicolor*). In contrast, the seeds ranged from 74.8 mg/100 g dry weight (*H. nitida*) to 560.3 mg/100 g dry weight (*T. speciosum*). On the other hand, the concentration of malic acid in the mucilage ranged from 24.0 mg/100 g *w*/*w* (*T. bicolor*) to 2039.4 mg/100 g *w*/*w* (*T. speciosum*). Furthermore, the concentration of tartaric acid in mucilage ranged from 28.1 mg/100 g DW (*T. subincanum*) to 215.0 mg/100 g DW (*T. speciosum*). The seeds ranged from 3.9 mg/100 g DW (*H. nitida*) to 43.8 mg/100 g DW (*T. subincanum*). In this sense, the total organic acids, as a sum of the individual ones, showed a remarkable variation among the species studied, with significantly higher concentrations in the mucilage than in the seeds. In the mucilage, the concentrations ranged from 1697.4 mg/100 g DW (*T. bicolor*) to 2957.6 mg/100 g DW (*T. speciosum*), while in the seeds, they ranged from 94.6 (*H. nitida*) to 1150.7 mg/100 g DW (*T. subincanum*). Notably, the citric and malic acid values in *T. bicolor* mucilage were higher than those reported in Colombian varieties, with 72.0 mg/g DW and 32.0 mg/g DW, respectively [45].

The organic acids tested include citric, malic, and tartaric acids. In these cultivars, malic acid predominated over citric acid, contrasting with previous studies that identified citric acid as the major organic acid in *T. bicolor* [45]. These differences could be attributed to variations in cellular metabolism, as organic acids are products of the tricarboxylic acid (TCA) cycle, whose activity is influenced by factors such as cellular redox state, light and nutrient availability, and environmental conditions [46]. Organic acids also play an essential role in enhancing food flavour, maintaining nutritional value, and prolonging shelf life [47].

#### 3.2.3. Phenolic Compounds

The main phenolic compounds detected were gallic acid, 4-hydroxybenzoic acid, coumaric acid, syringic acid, chlorogenic acid, caffeic acid, naringenin, ferulic acid, rutin, kaemferol, quercetin glucoside, and quercetin.

The gallic acid content ranged from 6.6 mg/100 g dry weight in the mucilage and seeds of *T. subincanum* to 244.8 mg/100 g dry weight in the mucilage of *T. bicolor* and *H. nitida*. Concentrations of 4-hydroxybenzoic acid ranged from 54.8 mg/100 g *w*/*w* in the mucilage of *T. subincanum* to 2215.1 mg/100 g *w*/*w* in the mucilage of *T. bicolor*. Coumaric acid ranged from 300.4 mg/100 g DW in the mucilage of *T. speciosum* to 4485.6 mg/100 g DW in the mucilage of *T. subincanum*. Syringic acid was only detected in *T. subincanum* with values of 11.7 mg/100 g DW in the mucilage and 21.6 mg/100 g DW in the seeds. Chlorogenic acid content ranged from 14.3 mg/100 g DW in *H. nitida* seeds to 37.2 mg/100 g DW in *T. subincanum* seeds. Concentrations of caffeic acid ranged from 75.2 mg/100 g DW in *T. subincanum* mucilage to 6378.0 mg/100 g DW in *H. nitida* seeds.

Naringenin and ferulic acid were present only in the mucilage, with 445.1 mg/100 g DW in *T. speciosum* and 490.2 mg/100 g DW in *T. subincanum*, respectively. Rutin was only detected in the seeds of *T. subincanum* (38.1 mg/100 g DW) and *H. nitida* (42.0 mg/100 g DW). Kaempferol and quercetin glucoside were found exclusively in the mucilage of *T. subincanum* (6.9 mg/100 g DW and 7.1 mg/100 g DW, respectively) and the seeds of *H. nitida* (27.9 mg/100 g DW and 13.0 mg/100 g DW, respectively). Finally, quercetin ranged from 6.7 mg/100 g DW in *T. subincanum* mucilage to 286.8 mg/100 g DW in H. nitida seeds. Concentrations of total phenolics, as the sum of individual compounds, ranged from 18.7 mg/100 g DW in *H. nitida* to 5251.9 mg/100 g DW in *T. bicolor* in the mucilage, and from 0.1 mg/100 g DW in *T. speciosum* to 7006.3 mg/100 g DW in *H. nitida* seeds.

These results agree with previous studies that reported higher concentrations of phenolic compounds in *T. subincanum* compared to *T. bicolor* [16]. In addition, other studies have shown the presence of chlorogenic acid, naringenin, syringic acid, caffeic acid, *p*-coumaric acid, *p*-hydroxybenzoic acid, and gallic acid in other cocoa species grown in Ecuador [18]. *m*-Coumaric acid is associated with benefits in controlling hyperglycaemia and improving antioxidant activity, which is essential in managing diabetes [48]. Caffeic acid is known for its antioxidant, anti-inflammatory, and anticarcinogenic properties [49,50]. In addition, 4-hydroxybenzoic acid, present in *T. bicolor*, was linked to the restoration of CoQ10 biosynthesis in CoQ10-deficient cells, highlighting its relevance to patients with CoQ10-related disorders [51].

Total phenolic content ranged from 0.1 mg/100 g DW in *T. speciosum* seeds to 5251.9 mg/100 g DW in *T. bicolor* mucilage. These values exceed those reported in previous studies, such as 5.0 mg/g DW in *T. bicolor* and 3.2 mg/g DW in *T. speciosum* [52,53] and a concentration of 55.2 mg/g DW in *T. subincanum* [52]. However, other studies have reported higher levels of total phenolics in mucilage than in the present study, with 169.0 mg/g DW in *T. bicolor* [45].

#### 3.2.4. Carotenoids

As for the carotenoids, the main molecules such as lutein, zeaxanthin, α-carotene, and β-carotene were identified. Lutein showed concentrations ranging from 0.1 mg/100 g DW in the mucilage and seeds of *T. subincanum* and *H. nitida* to 3.7 mg/100 g DW in the seeds of *T. bicolor*. Zeaxanthin was found in both the mucilage (0.6 mg/100 g DW) and seeds (0.2 mg/100 g DW) of *T. bicolor*. α-Carotene was only present in *H. nitida* seeds (0.1 mg/100 g DW), while β-Carotene showed concentrations ranging from 0.2 mg/100 g DW in *T. speciosum* to 5.1 mg/100 g DW in *T. bicolor*. Total carotenoids ranged from 0.2 in *T. speciosum* seeds to 6.0 mg/100 g DW in *T. bicolor*. Other studies have reported the presence of zeinoxanthin in *H. nitida* seeds [18]. In addition to its antioxidant properties, lutein is associated with benefits for eye health, cognitive function, and prevention of chronic diseases such as cancer [54].

#### 3.2.5. Chlorophyll and Derivatives

Chlorophyll and its derivatives were detected exclusively in seeds, with pheophytin being the predominant compound. Chlorophyll b showed concentrations ranging from 0.1 mg/100 g DW in *H. nitida* to 11.5 mg/100 g DW in *T. bicolor*. Pheophytin a was only present in *T. subincanum* seeds, whereas pheophytin b was found in *T. subincanum* (19.1 mg/100 g DW) and *T. bicolor* (33.8 mg/100 g DW). Chlorophyll and its derivatives showed a concentration range between 0.1 mg/100 g DW in *H. nitida* and 45.3 mg/100 g DW in *T. bicolor*. Thus, the degradation of chlorophyll during ripening and postharvest storage leads to the formation of pheophytin, a key process in fruit colour change [55]. This suggests that *T. subincanum* and *T. bicolor* seeds may not have reached full maturity.

### 3.3. Antioxidant Activity Analyses

Table 3 shows the average values of antioxidant activity, evaluated by the DPPH and ABTS methods, in the cocoa species studied. Using the DPPH method, the antioxidant activity in the mucilage ranged from 2.3 mmol TE/100 g DW (*T. bicolor*) to 5.8 mmol TE/100 g DW (*T. speciosum*). In seeds, the values ranged from 1.0 mmol TE/100 g DW in *T. speciosum* to 4.5 mmol TE/100 g DW in *T. bicolor*. In mucilage, antioxidant activity by the ABTS method ranged from 3.3 mmol TE/100 g DW in *T. bicolor* to 5.7 mmol TE/100 g DW in *T. speciosum*. In seeds, the values ranged from 1.3 mmol TE/100 g DW in *T. speciosum* to 5.7 mmol TE/100 g DW in *T. bicolor*. Overall, the antioxidant activity showed significant differences between mucilage and seeds, with higher levels in seeds. This agrees with previous studies on *H. nitida* seeds, where an antioxidant activity of 20.1 µmol ascorbic acid eq/g DW was reported [18].

Although no specific data are available on the antioxidant activity of the species studied, research on cocoa (*T. cacao*) by-products, such as mucilage, has shown elevated levels of polyphenols that contribute significantly to their antioxidant capacity. For example, the mucilage of the Nacional × Trinitario variety contains 105.8 mg gallic acid equivalent (GAE)/100 mL. In contrast, the mucilage of the CCN-51 variety showed values of 4.69 µM TE/mL compared to 8.54 µM TE/mL in other species [56].

Significantly, its antioxidant activity can vary considerably depending on the variety, processing methods, and the presence of other bioactive compounds that modulate its overall antioxidant capacity. Polyphenols, particularly flavonoids, play an essential role in these properties and their interaction with other compounds, such as vitamin C, can enhance their antioxidant benefits. Combining vitamin C and phenolic compounds in cocoa may provide additional health benefits. This interaction was shown to improve lipid profiles and reduce markers of oxidative damage, thereby promoting cardiovascular health. In particular, cocoa flavonoids, in synergy with vitamin C, help to reduce oxidative stress and inflammation, key factors in developing cardiovascular disease and cardiometabolic disorders [57,58].

On the other hand, in vitro tests used to evaluate antioxidant activity often do not accurately predict the behaviour of antioxidants in in vivo biological systems. This is due to differences in the bioavailability, metabolism, and interaction of these compounds in living organisms. In addition, in vitro assays present a significant problem of reproducibility, as variations in experimental protocols can lead to inconsistent results between studies. Therefore, although in vitro tests are valuable tools for the initial detection and mechanistic analysis of antioxidant activity, it is essential to complement them with in vivo studies to obtain a more complete assessment of their efficacy and health implications, such as specific cell lines, cell co-cultures, and 3D systems such as organoids or animal models [59,60].

### 3.4. Antimicrobial Activity Analyses

**Figure 2:**
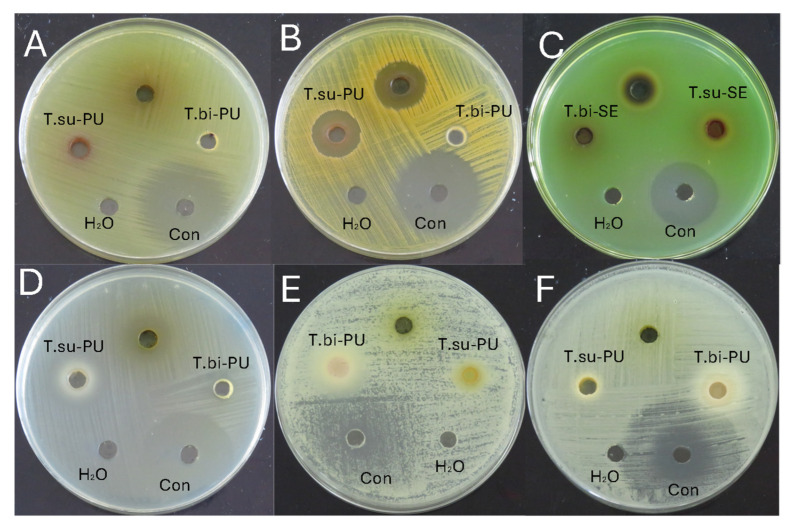
Antimicrobial activity of cocoa species: (**A**) Escherichia coli; (**B**) Staphylococcus aureus; (**C**) Pseudomonas aeruginosa; (**D**) Streptococcus mutans; (**E**) Candida albicans; and (**F**) Candida tropicalis. Note: T.su-PU, pulp of *T. subincanum*; T.bi-PU, pulp of *T. bicolor*; T.su-SE, seeds of *T. subincanum*; T.bi-PU, seeds of *T. bicolor*; Con, control.

Table 4 shows the average values of the antimicrobial activity of the cocoa species studied and Figure 2 an example of the antimicrobial activity test. Table 5 shows the minimum inhibitory concentration for the microorganisms that showed activity. The antibacterial activity was evaluated against *Escherichia coli, Staphylococcus aureus, Pseudomonas aeruginosa*, and *Streptococcus mutans*. The activity was observed only against *S. aureus* and *S. mutans*. In the case of *S. aureus*, inhibition halos were observed in the mucilage and seeds of *T. subincanum* and the seeds of *T. bicolor*. On the other hand, activity against *S. mutans* was observed in the mucilage of *T. subincanum* and the seeds of *T. bicolor*. The antifungal activity was evaluated against *Candida albicans* and *Candida tropicalis*. The results showed activity only on the seeds of *T. bicolor.*

Although no previous specific studies on the antimicrobial activity of these species were found, cocoa (*T. cacao*) is known to have excellent antimicrobial activity against a wide range of microorganisms due to its complex chemical composition. For example, the ethanolic extract of cocoa beans was shown to have antimicrobial properties against *Streptococcus mutans*, a bacterium associated with dental caries. However, it is less effective than chlorhexidine, a standard antimicrobial used in dentistry [61]. Similarly, aqueous and methanolic leaf extracts of cocoa have shown activity against *Staphylococcus aureus*, with inhibition zones ranging from 12.0 to 26.3 mm at 100 to 500 mg/mL concentrations, indicating a remarkable antimicrobial potential [62].

In addition, organic acids present in these strains may contribute to their antimicrobial activity. These compounds alter the bacterial balance and inhibit the enzymatic activity of microorganisms, thereby reducing harmful bacteria in the gut and other environments [47].

On the other hand, the limited antimicrobial activity observed in the study against *E. coli* and *P. aeruginosa* could be attributed to a combination of factors, including the robust structure of the cell wall and an outer membrane of large negative bacteria, the presence of highly efficient efflux pumps, the formation of biofilms, and remarkable genetic plasticity. These mechanisms not only hinder the action of natural compounds but also explain the intrinsic resistance of these bacteria to a wide range of antimicrobial agents [63,64]. In addition, although conventional in vitro methods are still widely used to assess antimicrobial activity, technological advances have enabled the development of new techniques with greater sensitivity, reproducibility, and analytical efficiency, such as ex vivo systems, cell lines, or microbiota models. However, the high cost and limited accessibility of these methods limit their widespread use, especially in resource-limited regions [65].

### 3.5. Statistical Analysis

**Figure 3:**
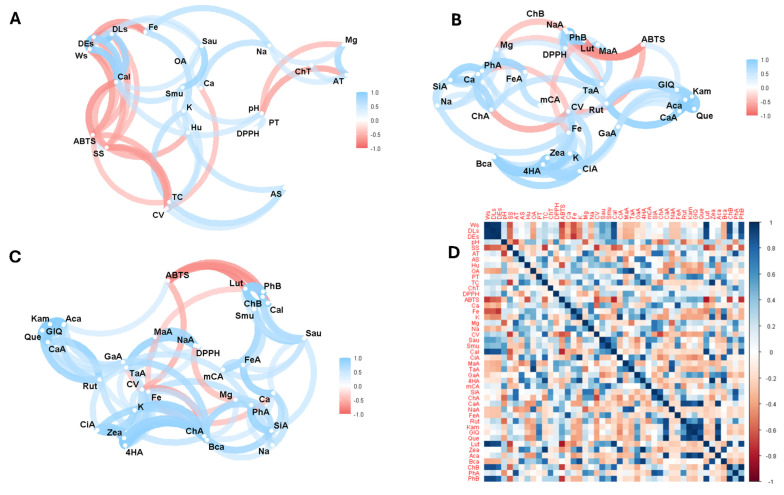
Exploratory multivariate analysis using Pearson correlation. (**A**) All variables including total bioactive compounds; (**B**) bioactive compounds and antioxidant activity; (**C**) bioactive compounds, antioxidant, and antimicrobial activity; (**D**) correlation matrix considering all variables in study. Note: Ws, seed weight; DLs, seed longitudinal diameter; DEs, seed equatorial diameter; SS, soluble solids; AT, total titratable acidity; AS, ash; Hu, humidity; OA, total organic acid; PT, total phenolics; TC, total carotenoids; ChT, total chlorophyll and their derivatives; DPPH, antioxidant activity by DPPH; ABTS, antioxidant activity by ABTS; Ca, calcium; Fe, iron; K, potassium; Mg, magnesium; Na, sodium; CV, vitamin C; Sau, *Staphylococcus aureus*; Smu, *Streptococcus mutans*; Cal, *Candida albicans*; CiA, citric acid; MaA, malic acid; TaA, tartaric acid; GaA, galic acid; 4HA, 4-hydroxybenzoic acid; mCA, *m*-coumaric acid; SiA, syringic acid; ChA, chlorogenic acid; CaA, caffeic acid; NaA, naringenin; FeA, ferulic acid; Rut, rutin; Kam, kaempferol; GIQ, quercetin glucoside; Que, quercetin; Lut, lutein; Zea, zeaxanthin; Aca, α-carotene; Bca, β-carotene; ChB, chlorophyll b; PhA, pheophytin a; PhB, pheophytin b.

Figure 3 illustrates the Pearson correlation analysis between the variables studied. Thus, Figure 3A shows the correlation analysis considering the physico-chemical properties, mineral content, vitamin C, antioxidant activity, antimicrobial activity, and total phenolics, carotenoids, and organic acids. Figure 3B shows the analysis focusing on minerals, vitamin C, antioxidant activity, and individual compounds of organic acids, phenols, and carotenoids. Figure 3C shows the correlation analysis of all variables except physical and chemical properties such as seed weight and size, pH, soluble solids, titratable acidity, moisture, and ash. Finally, Figure 3D shows the complete correlation matrix considering all the variables studied.

Correlation analysis showed a direct relationship between weight and equatorial seed diameter with antifungal activity against *Candida albicans*, iron with sodium and total organic acids; *S. aureus* with *S. mutans*; and Mg with titratable acidity, total phenolics with antioxidant activity by the DPPH method, pH with moisture. Thus, studies on plant species have demonstrated a correlation between total phenols and antioxidant activity evaluated by the DPPH method [18,24].

On the other hand, an inverse relationship was found between vitamin C and soluble solids, total carotenoids with antioxidant activity according to the ABTS method, weight with antioxidant activity, antioxidant activity with antifungal activity against *Candida albicans*, and total chlorophyll and its derivatives with pH and titratable acidity. Thus, *T. bicolor* is the cocoa variety with the largest size and weight compared to the other species. Several genetic and environmental factors influence the relationship between cocoa weight and size. Genetic studies have shown that traits such as length, seed width, and seed length/width ratio are polygenic, meaning that they are controlled by several genes located on different chromosomes. Environmental factors such as soil composition and fertilisation also play an essential role in determining the growth and development of cocoa plants, affecting the size and weight of cocoa seeds [66,67]. An inverse relationship was observed between soluble solids and cocoa seed size. This finding is described in the literature as one where larger fruits accumulate more soluble solids but have lower soluble solids content compared to small fruits. This is attributed to the dilution effect due to larger fruits’ greater volume of liquid [68].

In the case of bioactive compounds, a direct relationship was observed between 4-hydroxybenzoic acid and total carotenoids; potassium and *Candida albicans*; *m*-coumaric acid with *S. mutans* inhibition; ferulic acid with antioxidant activity by ABTS; rutin, kaempferol, quercetin glycoside, and quercetin with *C. albicans* inhibition; lutein with weight, size, and *C. albicans* inhibition; β-carotene with weight and size; chlorophyll with weight and *C. albicans* inhibition; and pheophytin B with weight and *C. albicans* inhibition. Several of these correlations were observed in other studies [23,24].

**Figure 4:**
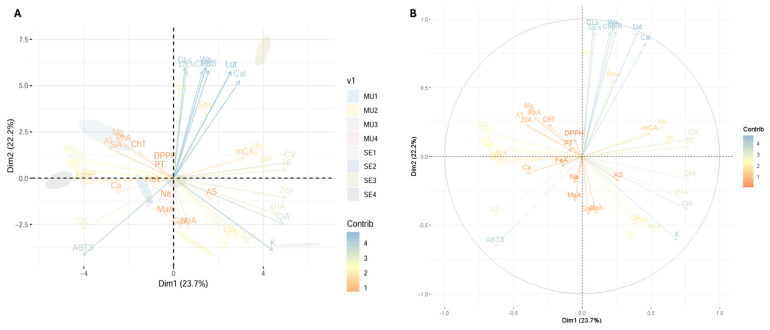
Exploratory multivariate analysis using principal component. (**A**) PCA with distribution of fractions under study; (**B**) contribution of each variable to PCA. Note: Ws, seed weight; DLs, seed longitudinal diameter; DEs, seed equatorial diameter; SS, soluble solids; AT, total titratable acidity; AS, ash; Hu, humidity; OA, total organic acid; PT, total phenolics; TC, total carotenoids; ChT, total chlorophyll and their derivatives; DPPH, antioxidant activity by DPPH; ABTS, antioxidant activity by ABTS; Ca, calcium; Fe, iron; K, potassium; Mg, magnesium; Na, sodium; CV, vitamin C; Sau, *Staphylococcus aureus*; Smu, *Streptococcus mutans*; Cal, *Candida albicans*; CiA, citric acid; MaA, malic acid; TaA, tartaric acid; GaA, galic acid; 4HA, 4-hydroxybenzoic acid; mCA, *m*-coumaric acid; SiA, syringic acid; ChA, chlorogenic acid; CaA, caffeic acid; NaA, naringenin; FeA, ferulic acid; Rut, rutin; Kam, kaempferol; GIQ, quercetin glucoside; Que, quercetin; Lut, lutein; Zea, zeaxanthin; Aca, α-carotene; Bca, β-carotene; ChB, chlorophyll b; PhA, pheophytin a; PhB, pheophytin b; MU1, mucilage *T. subincanum*; MU2, mucilage *T. speciososum*; MU3, mucilage *T. bicolor*; MU4, mucilage *H. nitida*; SE1, seed *T. subincanum*; SE2, seed *T. speciososum*; SE3, seed *T. bicolor*; SE4, seed *H. nitida*.

Figure 4 shows the principal component analysis (PCA) performed on the variables studied in the cocoa species. This analysis makes it possible to visualise the grouping patterns of the samples and the relationships between the variables studied, providing a comprehensive representation of the variability in the data. For example, Figure 4A shows the PCA together with the distribution of the samples analysed, distinguishing between slime and seeds. Figure 4B shows the distribution of the variables in the principal component space and the relative contribution of each variable to the variance explained by each principal component.

The principal component analysis shows that the largest source of variability in the data is 23.7% for Dim1, while Dim2 showed a value of 22.2%. Thus, variables such as Ca, Na, antioxidant activity, and total phenolics are more associated with the second quadrant, indicating that these characteristics are related and contribute significantly to the variability explained by Dim2. On the other hand, variables such as K, pH, and carotenoids such as lutein and zeaxanthin are grouped in the first quadrant, indicating that they are more associated with *T. bicolor* seeds.

## 4. Conclusions

This study on the bioactive compounds, antioxidants, and antimicrobial activity of the seeds and pulp of non-traditional cocoa species (*Theobroma subincanum, T. speciosum, T. bicolor*, and *Herrania nitida*) showed differentiated profiles of bioactive compounds, with significant contents of minerals, vitamin C, phenols, carotenoids, and organic acids. *Theobroma subincanum* seeds stood out for their high content of titratable acidity, magnesium, sodium, syringic acid, chlorogenic acid, caffeic acid, rutin, and quercetin. The mucilage was noted for its calcium content, *m*-coumaric acid, ferulic acid, kaempferol, quercetin glycoside, and antimicrobial activity against *Streptococcus mutans*. The mucilage of *T. speciosum* showed the highest levels of malic acid, tartaric acid, naringenin, and antioxidant activity by DPPH and by ABTS. In *Theobroma bicolor*, the seeds were noted for their high pH, lutein content, and antimicrobial activity against *S. aureus* and *C. albicans*. The mucilage, on the other hand, showed high levels of iron, potassium, vitamin C, citric acid, gallic acid, 4-hydroxybenzoic acid, zeaxanthin, β-carotene, and antioxidant activity by ABTS. Finally, *H. nitida* mucilage showed high soluble solids levels. The results support that these non-traditional cocoa species are a valuable source of bioactive compounds that contribute significantly to antioxidant and antimicrobial activity, opening new opportunities for sustainable use and integration into value-added products. Furthermore, these findings highlight the need for studies using in vivo models to more accurately assess their impact on human health and their potential application in the food and pharmaceutical industries.

## Figures and Tables

**Table 1:** Average values of fruit and seed weights of cocoa species.

	*Theobroma subincanum*			*Theobroma speciosum*			*Theobroma bicolor*			*Herrania nitida*		
**Fruit**												
Weight (g)	159.1	±	28.5 ^b^	98.9	±	2.1 ^c^	2066.7	±	57.7 ^a^	36.7	±	0.3 ^d^
Longitudinal diameter (mm)	78.5	±	18.8 ^c^	21.8	±	0.9 ^d^	470.0	±	1.7 ^a^	93.3	±	0.2 ^b^
Equatorial diameter (mm)	65.1	±	3.3 ^b^	12.4	±	0.2 ^d^	303.3	±	0.0 ^a^	47.2	±	0.1 ^c^
**Seeds**												
Weight (g)	1.9	±	0.1 ^b^	1.6	±	0.1 ^c^	5.4	±	0.7 ^a^	1.2	±	0.0 ^d^
Longitudinal diameter (cm)	0.8	±	0.2 ^b^	0.3	±	0.1 ^c^	1.9	±	0.1 ^a^	0.8	±	0.1 ^b^
Equatorial diameter (cm)	0.5	±	0.0 ^c^	0.4	±	0.0 ^d^	1.6	±	0.2 ^a^	0.8	±	0.1 ^b^

Note: Mean values ± SD. The lower-case letters next to the standard deviation indicate the separation of the mean values at a 95% confidence level between cocoa species.

**Table 2:** Average values of chemical characteristics of cocoa species.

		*Theobroma subincanum*						*Theobroma speciosum*						*Theobroma bicolor*						*Herrania nitida*							
		Mucilage			Seeds			Mucilage			Seeds			Mucilage			Seeds			Mucilage			Seeds			Am	As
pH		5.7	±	0.5 ^a^	3.7	±	0.0 ^b^	6.4	±	0.0 ^a^	3.7	±	0.1 ^b^	5.5	±	0.1 ^b^	6.7	±	0.1 ^a^	4.6	±	0.0 ^a^	4.2	±	0.0 ^b^	***	***
Soluble solids (°Brix)		10.3	±	0.6 ^a^	9.0	±	1.0 ^a^	8.9	±	0.6 ^a^	8.7	±	0.6 ^a^	7.7	±	1.2 ^a^	6.0	±	0.0 ^a^	12.0	±	0.0	10.0	±	1.0	***	**
Total titratable acidity (%)		0.6	±	0.1 ^b^	1.7	±	0.1 ^a^	0.1	±	0.0 ^b^	0.7	±	0.1 ^a^	0.6	±	0.0 ^a^	0.4	±	0.2 ^a^	0.5	±	0.2 ^a^	0.8	±	0.3 ^a^	**	***
Humidity (%)		84.0	±	6.4	61.8	±	11.5	39.5	±	5.6 ^a^	39.3	±	5.6 ^a^	96.0	±	0.2 ^a^	92.7	±	0.4 ^b^	81.3	±	2.5 ^a^	37.6	±	5.9 ^b^	***	***
Ash (%)		1.4	±	0.2 ^b^	2.6	±	0.3 ^a^	1.2	±	0.2 ^b^	2.5	±	0.5 ^a^	4.2	±	0.3 ^a^	1.7	±	0.1 ^b^	1.2	±	0.2 ^b^	2.6	±	0.3 ^a^	***	*
Mineral Profile (mg/100 g DW)	Ca	1304.1	±	35.9 ^a^	752.1	±	57.6 ^b^	432.5	±	8.2 ^a^	126.4	±	10.0 ^b^	276.4	±	42.0 ^a^	129.0	±	3.9 ^b^	321.3	±	2.5 ^b^	409.3	±	54.7 ^a^	***	***
	Fe	52.4	±	3.9 ^a^	44.6	±	7.0 ^a^	46.8	±	4.4 ^a^	12.7	±	0.3 ^b^	77.0	±	7.2 ^a^	14.0	±	0.4 ^b^	49.7	±	0.8 ^a^	12.4	±	1.3 ^b^	*	**
	K	1342.6	±	65.5 ^a^	1229.2	±	68.7 ^a^	2155.3	±	19.5 ^a^	1896.6	±	13.8 ^b^	4595.2	±	162.5 ^a^	999.7	±	53.3 ^b^	1523.1	±	17.4 ^a^	774.4	±	11.4 ^b^	***	***
	Mg	314.6	±	28.2 ^a^	382.7	±	0.8 ^a^	160.7	±	17.0 ^b^	313.6	±	2.0 ^a^	250.7	±	39.6 ^a^	238.7	±	4.0 ^a^	190.3	±	2.5 ^b^	280.2	±	4.1 ^a^	*	***
	Na	54.8	±	3.1 ^a^	59.3	±	1.9 ^a^	8.8	±	1.9 ^a^	2.7	±	0.2 ^b^	54.1	±	2.7 ^a^	5.2	±	1.3 ^b^	18.1	±	0.2 ^a^	16.7	±	3.7 ^a^	***	***

Note: Mean values ± SD. The lower-case letters next to the standard deviation indicate the separation of the mean values at a 95% confidence level between the seeds and mucilage. The significance of differences between the mucilage of cocoa species (Am) and the significance of differences between the seeds of cocoa species (As) is given ns, not significant; *, *p* < 0.1; **, *p* < 0.01; ***, *p* < 0.001.

**Table 3:** Average values of vitamin C, organic acids, phenolics, carotenoids, and antioxidant activity of cocoa species.

		*Theobroma subincanum*						*Theobroma speciosum*						*Theobroma bicolor*						*Herrania nitida*							
		Mucilage			Seeds			Mucilage			Seeds			Mucilage			Seeds			Mucilage			Seeds			Am	As
Vitamin C (mg/100 g DW)		3.81	±	0.0	n	d		3.9	±	0.1 ^b^	55.1	±	2.5 ^a^	70.5	±	1.8 ^a^	54.4	±	3.6 ^b^	nd			0.9	±	0.1	***	***
Organic Acids (mg/100 g DW)	Citric acid	112.4	±	4.6 ^b^	425.8	±	43.0 ^a^	703.2	±	129.1 ^a^	560.3	±	23.9 ^a^	1557.9	±	34.4 ^a^	431.4	±	27.8 ^b^	120.1	±	12.1 ^a^	74.8	±	1.8 ^b^	***	***
	Malic acid	1112.8	±	183.3 ^a^	681.1	±	123.0 ^a^	2039.4	±	365.8 ^a^	472.9	±	11.1 ^b^	24.0	±	1.1 ^b^	139.0	±	15.0 ^a^	62.1	±	2.5 ^a^	16.0	±	0.2 ^b^	**	**
	Tartaric acid	28.1	±	16.0 ^a^	43.8	±	8.9 ^a^	215.0	±	35.7 ^a^	18.5	±	0.8 ^b^	115.4	±	8.8 ^a^	34.8	±	3.3 ^a^	12.1	±	1.2 ^a^	3.9	±	0.1 ^b^	**	**
	Total acids	1253.3	±	203.9 ^a^	1150.7	±	174.9 ^a^	2957.6	±	530.5 ^a^	1051.7	±	35.8 ^b^	1697.4	±	44.3 ^a^	605.2	±	16.0 ^a^	194.3	±	11.5 ^a^	94.6	±	1.5 ^b^	*	***
Phenolics Profile (mg/100 g DW)	Galic acid	6.6	±	0.2 ^a^	6.6	±	0.0 ^a^	54.8	±	1.3				244.8	±	0.6							244.4	±	0.9	***	***
	4-hydroxybenzoic acid	54.8	±	0.8										2215.1	±	104.0 ^a^	361.0	±	38.8 ^b^							***	-
	*m*-Coumaric acid	4485.6	±	235.0				300.4	±	5.3				2792.1	±	114.5 ^a^	3045.5	±	164.0 ^a^							***	-
	Syringic acid	11.7	±	0.4 ^b^	21.6	±	1.0 ^a^																			-	-
	Chlorogenic acid	16.1	±	0.1 ^b^	37.2	±	3.3 ^a^													18.7	±	0.2 ^a^	14.3	±	0.1 ^b^	-	***
	Caffeic acid	75.2	±	6.2 ^b^	2223.0	±	43.5 ^a^																6378.0	±	581.5	-	***
	Naringenin							445.1	±	5.2																-	-
	Ferulic acid	490.2	±	53.4																						-	-
	Rutin				38.1	±	2.3																42.0	±	0.3	-	***
	Kaempferol	6.9	±	0.7																			27.9	±	4.0	-	-
	Quercetin glucoside	7.1	±	0.7																			13.0	±	0.1	-	-
	Quercetin	6.7	±	0.9 ^b^	71.9	±	2.8 ^a^																286.8	±	0.1	-	***
	Total phenolics	5161.0	±	282.8 ^a^	2398.4	±	39.7 ^b^	800.4	±	1.4 ^a^	0.1	±	0.0 ^b^	5251.9	±	217.8 ^a^	3406.5	±	202.9 ^b^	18.7	±	0.2 ^b^	7006.3	±	578.8 ^a^	***	**
Carotenoids profile (mg/100 g DW)	Lutein	0.1	±	0.0 ^b^	1.1	±	0.1 ^a^				0.2	±	0.0	0.3	±	0.1 ^b^	3.7	±	0.1 ^a^	0.1	±	0.0	0.1	±	0.0	ns	***
	Zeaxanthin													0.6	±	0.0 ^a^	0.2	±	0.0 ^b^							-	-
	α-carotene																						0.1	±	0.0	-	-
	β-carotene	0.8	±	0.1				1.0	±	0.0				5.1	±	0.1							1.1	±	0.2	***	-
	Total Carotenoids	0.9	±	0.1 ^b^	1.1	±	0.1 ^a^	1.01	±	0.0 ^a^	0.2	±	0.0 ^b^	6.0	±	0.0 ^a^	3.9	±	0.1 ^b^	0.1	±	0.0 ^b^	1.3	±	0.2 ^a^	***	***
Chlorophylls and their derivatives (mg/100 g DW)	Chlorophyll b				7.8	±	0.1				0.4	±	0.1				11.5	±	0.4	0.1	±	0.0				-	***
	Pheophytin a				2.5	±	0.0																			-	-
	Pheophytin b				19.1	±	2.5										33.8	±	2.7							-	*
	Total chlorophyll																			0.1	±	0.0					
**Antioxidant activity DDPH** (mmol TE/100 g DW)	DPPH	3.6	±	0.2 ^a^	3.7	±	0.6 ^a^	5.8	±	0.5 ^a^	1.0	±	0.3 ^b^	2.3	±	0.1 ^b^	4.5	±	0.2 ^a^	2.8	±	0.3 ^b^	3.8	±	0.2 ^a^	***	***
	ABTS	4.9	±	0.4 ^a^	4.0	±	0.6 ^b^	5.7	±	0.6 ^a^	1.3	±	0.2 ^b^	3.3	±	0.2 ^b^	5.7	±	0.2 ^a^	3.9	±	0.5 ^a^	3.9	±	0.4 ^a^	***	***

Note: Mean values ± SD. The lower-case letters next to the standard deviation indicate the separation of the mean values at a 95% confidence level between the seeds and mucilage. The significance of differences between the mucilage of cocoa species (Am) and the significance of differences between the seeds of cocoa species (As) is given ns, not significant; *, *p* < 0.1; **, *p* < 0.01; ***, *p* < 0.001; nd, not detectable.

**Table 4:** Average values of zone of inhibition of cocoa species extract against bacteria and fungi.

Zone of Inhibition (mm)																				
		*Theobroma subincanum*						*Theobroma speciosum*		*Theobroma bicolor*						*Herrania nitida*		*Control*		
		Mucilage			Seeds			Mucilage	Seeds	Mucilage			Seeds			Mucilage	Seeds			
Bacterial strain	*E. coli* ATCC 8739	-			-			-	-	-			-			-	-	25.3	±	1.3
	*S. aureus* ATCC 6538P	17.0	±	0.0	13.0	±	1.4	-	-	-			17.5	±	0.7	-	-	27.0	±	2.2
	*P. aeruginosa* ATCC 9027	-			-			-	-	-			-			-	-	24.5	±	1.3
	*S. mutans* ATCC 25175	17.0	±	1.4	-			-	-	-			16.0	±	0.0	-	-	30.3	±	1.0
Fungal strain	*C. albicans* ATCC 1031	-			-			-	-	-			10.0	±	0.0	-	-	11.0	±	0.5
	*C. tropicalis* ATCC 13803	-			-			-	-	-			-			-	-	17.0	±	1.9

Note: Mean values + SD.

**Table 5:** Average values of minimum inhibitory concentration.

		Minimum Inhibitory Concentration (mg/mL)								
		*S. aureus* ATCC 6538P			*S. mutans* ATCC 25175			*C. albicans* ATCC 1031		
*Theobroma subincanum*	Mucilage	37.5	±	0.0	37.5	±	0.0			
	Seeds	18.8	±	0.0						
*Theobroma bicolor*	Seeds	19.1	±	0.4	75.0	±	0.0	75.0	±	0.0

## Data Availability

The original contributions presented in this study are included in the article. Further inquiries can be directed to the corresponding author.
